# Oncolytic Avian Reovirus σA-Modulated Upregulation of the HIF-1α/C-myc/glut1 Pathway to Produce More Energy in Different Cancer Cell Lines Benefiting Virus Replication

**DOI:** 10.3390/v15020523

**Published:** 2023-02-13

**Authors:** Chao-Yu Hsu, Jing-Wen Huang, Wei-Ru Huang, I-Chun Chen, Ming-Shan Chen, Tsai-Ling Liao, Yu-Kang Chang, Muhammad Munir, Hung-Jen Liu

**Affiliations:** 1 Division of Urology, Department of Surgery, Tungs’ Taichung MetroHarbor Hospital, Taichung 435, Taiwan; 2 Ph.D Program in Translational Medicine, National Chung Hsing University, Taichung 402, Taiwan; 3 Institute of Molecular Biology, National Chung Hsing University, Taichung 402, Taiwan; 4 The iEGG and Animal Biotechnology Center, National Chung Hsing University, Taichung 402, Taiwan; 5 Department of Psychiatry, Taichung Veterans General Hospital, Taichung 407, Taiwan; 6 Department of Anesthesiology, Ditmanson Medical Foundation Chia-Yi Christian Hospital, Chia-Yi 600, Taiwan; 7 Department of Medical Research, Taichung Veterans General Hospital, Taichung 407, Taiwan; 8 Department of Medical Research, Tungs’ Taichung MetroHarbor Hospital, Taichung 435, Taiwan; 9 Department of Post-Baccalaureate Medicine, National Chung Hsing University, Taichung 402, Taiwan; 10 Department of Biomedical and Life Sciences, Lancaster University, Lancashire LA1 4YW, UK; 11 Rong Hsing Research Center for Translational Medicine, National Chung Hsing University, Taichung 402, Taiwan; 12 Department of Life Sciences, National Chung Hsing University, Taichung 402, Taiwan

**Keywords:** avian reoviruses, oncolytic virus, glycolysis, c-myc, HIF-1α, glut1, ATeams

## Abstract

Our previous reports proved that the structural protein σA of avian reovirus (ARV) is an energy activator which can regulate cellular metabolism that is essential for virus replication. This study has further demonstrated that the ARV protein σA is able to upregulate the HIF-1α/myc/glut1 pathway in three cancer cell lines (A549, B16-F10, and HeLa) to alter the metabolic pathway of host cells. Quantitative real-time RT-PCR and Western blotting results have revealed that σA protein could enhance both mRNA and the protein levels of HIF-1α, c-myc, and glut1 in these cancer cell lines. In this work, ATeam immunofluorescence staining was used to reveal that knockdown of HIF-1α, c-myc, and glut1 by shRNAs decreased cellular ATP levels. Our data reveal that the ARV σA protein can downregulate lactate fermentation and upregulate glutaminolysis. The σA protein upregulates glutaminase, which converts glutamate into the TCA cycle intermediate α-ketoglutarate, activating the TCA cycle. In the lactate fermentation pathway, ARV σA protein suppresses lactate dehydrogenase A (LDHA), implying the Warburg effect does not occur in these cancer cell lines. This study provides a novel finding revealing that ARV σA protein upregulates glycolysis and glutaminolysis to produce energy using the HIF-1α/c-myc/glut1 pathway to benefit virus replication in these cancer cell lines.

## 1. Introduction

According to the World Health Organization (WHO) statistics, cancer is the second highest cause of death in the world. Recently, the International Agency for Research on Cancer (IARC) of the WHO released the latest global cancer data for 2020, with a total of 19.29 million new cancer cases and 9.96 million deaths worldwide [1]. Extensive cancer treatment methods include chemotherapy, targeted drugs, and immunotherapy; but these therapies generally have disadvantages such as high prices, strong side effects, and resistance to anticancer drugs. Therefore, it is even more important to find new anticancer treatments. Oncolytic virotherapy is an emerging therapy that uses natural or re-edited viruses for cancer treatment. The virus needs to have the potential to target, inhibit and kill cancer cells. Such viruses are called oncolytic viruses [2]. To meet the needs of rapid proliferation and metastasis, cancer cells have evolved a set of unique metabolic pathways aimed at increasing the uptake and synthesis of nutrients such as carbohydrates, proteins, and fats [3]. A phenomenon like this is the metabolic reprogramming of cancer cells, including increased glycolysis, lactate fermentation, fatty acid metabolism, and glutamine metabolism [4]. The c-myc and hypoxia-inducible factor 1 (HIF-1α) are abnormally expressed in large quantities in human cancers. HIF-1α and c-myc cooperate to regulate glycolysis and stimulate the Warburg effect [5]. HIF-1α and c-myc have been shown to synergistically promote hexokinase 2 (HK2) and pyruvate dehydrogenase kinase 1 (PDK1), which in turn increase the conversion of glucose to lactate. HIF-1α and c-myc independently activate glucose transporter 1 (glut1) and lactate dehydrogenase A (LDHA). All the above metabolic pathways contribute to the Warburg effect [6].

Recent reports have confirmed that ARV has good oncolytic activity, can induce cancer cell apoptosis, promote cancer cell immune response and expose tumor-associated antigens to the immune system [7,8,9,10]. The nonstructural protein p17 of ARV can regulate many cellular signaling pathways, induce autophagy, and activate protein kinase R (PKR) [8]. Both signaling pathways activate innate immunity to induce an immune response against tumor cells, ultimately leading to cancer cell death [7]. Additional studies have proved that mammalian reovirus (MRV) type-3 is also an oncolytic virus, and the drug Reolysin isolated from this virus has been approved by the US Food and Drug Administration (FDA) in 2015 and 2017. An FDA license for the treatment of glioma and breast cancer has also been used in various cancer treatments [11,12]. Since MRV type 3 virus is a mammalian virus, antibodies may exist in the human body due to infection with the virus, which may easily lead to potential factors such as immune rejection of the virus by the human body. Given ARV is not a zoonotic virus and there is a lack of pre-existing immunity, ARV offers superior properties in cancer treatments. The structural protein σA encoded by the S2 gene of ARV is the inner coat protein of the virus [13]. It has been documented that the ARV proteins p17 and σA co-repress mTOR complex 2 and CDK2/cyclin A2 complex to induce autophagy. The p17 protein can positively regulate p53 to activate PTEN to inhibit the PI3K/Akt/mTORC1 pathway, thereby inducing autophagy to benefit virus replication [14,15]. ARV σA protein has been demonstrated to upregulate the expression of glutamate dehydrogenase (GDH), isocitrate dehydrogenase 3 subunit β (IDH3B) and HIF-1α in Vero cells, thereby activating glycolysis, promoting TCA cycle, and making host cells to produce more energy, which is conducive to virus replication and infection [16]. Since ARV can alter the metabolic pathways of the host cells to enhance virus replication, the aim of this study is to investigate whether the σA protein of ARV can upregulate glycolysis and TCA cycle of different cancer cell lines. This study demonstrates for the first time that σA upregulates the HIF-1α/myc/glut1 pathway which increases glutaminolysis and suppresses LDHA. This work provides valuable information for better insights into σA-modulated glycolysis and TCA cycle to increase intracellular ATP to promote virus replication and to compete for energy and material cell growth which is required by the cancer, and to finally achieve anti-cancer effects.

## 2. Materials and Methods

### 2.1. Virus and Cells

The S1133 strain of ARV has been used in this study. African green monkey (Vero) cell, melanoma cell (B16-F10), human fetal lung fibroblast 1 (HFL-1), human lung adenocarcinoma cell (A549), and human cervical cancer cell (HeLa) were cultured in minimum essential medium eagle (MEM), Dulbecco’s modified minimal essential medium (DMEM), or Kaighn’s modification of Ham’s F-12 medium (F12K) with 5–10% heat-inactivated fetal bovine serum (Hyclone, Logan, UT, USA) and 1% penicillin (100 IU/mL)/streptomycin (100 g/mL)(Gibco, Grand Island, NY, USA). Cells were propagated in a 37 °C, 5% CO_2_ humidified incubator. Cells were seeded one day before each experiment in 10-cm culture dishes and cultured until they reached about 75% confluence.

### 2.2. Antibodies

Mouse anti-c-myc monoclonal antibody was purchased from Santa Cruz Biotechnology (Dallas, TX, USA). Mouse anti-β-actin was purchased from Millipore Corporation (Billerica, MA, USA). Rabbit anti-HIF-1α, rabbit anti-glut1, rabbit anti-LDHA, rabbit anti-pyruvate kinase isozyme M2 (PKM2), rabbit anti-2-oxoglutarate dehyrogenase E1 component (OGDH), and rabbit anti-glutaminase (Gls) were purchased from Cell Signaling Technology (Danvers, MA, USA). Antibodies against ARV σA and p17 proteins were from our laboratory stocks [8,16]. The secondary antibodies, including goat anti-mouse IgG (H + L) and goat anti-rabbit IgG (H + L) HRP conjugate and rhodamine-labeled affinity purified antibody to mouse IgG (H + L) were purchased from Kirkegaard and Perry Laboratories (Washington, DC, USA).

### 2.3. shRNA and Plasmid Construction

The HIF-1α shRNA gene (catalog no. TRCN0000003808), c-myc shRNA gene (catalog no. TRCN0000174055), and glut1 shRNA gene (catalog no. TRCN0000151420) in the vector pLKO were obtained from the RNAi core facility (Academia Sinica, Taipei, Taiwan). The sequence for σA shRNA is as follows: GCGACGAATCGTACTCAATTA [16] and was obtained from OriGene Co. (Rockville, MD, USA). The pCI-neo-σA and pcDNA3.1-p17 plasmids have been described previously [8,17]. Cells were transfected with the respective shRNAs for 6 h followed by infection with ARV at an MOI 10 for 24 h. The whole cell lysates were collected for Western blot analysis.

### 2.4. Quantitative Real Time RT-PCR

To explore whether ARV infection and σA transfection affect the c-myc, HIF-1α, and glut1 of cancer cell lines (A549, B16-F10, and HeLa), cells were either infected with ARV at an MOI of 10 or transfected with the pCI-neo-σA plasmid. Cell lysates were collected at 24 h post infection. Total RNA was isolated from virus-infected or transfected cells using TRIzol reagent and subjected to quantitative real time RT-PCR with iQ^TM^ SYBR^®^ Green Supermix kit (Bio-Rad, Hercules, FL, USA) as described previously [16]. The specific primer pairs used in this work are shown in Table 1.

### 2.5. Transient Transfection

For the transfection experiments, cells were seeded into 6-well plates. At about 70% confluence, cells were transfected with the TranslT-X2 reagent (Mirus Bio, Madison, WI, USA) according to the manufacturer’s instruction. In this work, cancer cell lines were transfected with the respective shRNAs for the first 6 h followed by infection with ARV at an MOI of 10 for 24 h. The scramble plasmid was used as a negative control. Cell viability measurements are conducted following transfections with the respective genes or shRNAs. In order to ensure the transfection efficiency, it is mainly confirmed either using Western blot or immunofluorescence staining to ensure that the transfection efficiency reaches 80–90%.

### 2.6. Trypan Blue Dye Staining

In this study, 2.5 × 10^5^ cancer cells were seeded into 6-well culture plates. After transfection with the indicated plasmid, cells were collected using Trypsin-EDTA (Gibco, USA) and suspended in a culture medium. Viable and nonviable cells were determined using direct counting in the presence of 0.5% trypan blue using a hemocytometer [8].

### 2.7. Western Blot Assays

The cancer cell lines (A549, B16-F10, and HeLa) were seeded in 6-well culture plates one day before treating with infected virus or transfected plasmid as described above. The collected cells were washed twice with 1XPBS with lysis buffer (Cell signaling). The concentration of solubilized protein in cell lysates was determined using Bio-Rad Protein assay (Bio-Rad, USA). The sample was collected with 2.5× sample buffer dye and boiled in the water bath for 15 min. The samples were electrophoresed in 10–15% sodium dodecyl sulphate (SDS)-polyacrylamide gel and transferred to PVDF membrane (Amersham Biosciences, Little Chalfont, UK) for Western blot assay. Expression levels of each individual protein was examined using the respective primary antibody and horseradish peroxidase secondary antibody conjugate. After membrane incubation with enhanced chemiluminescence (ECL plus) reagent (Amersham Biosciences), the results were decocted on X-ray film (Kodak, Rochester, Rochester, NY, USA).

### 2.8. Determination of Virus Titer

The cancer cell lines HFL-1, A549, B16-F10, and HeLa were seeded in 4-well culture plates one day before infection with ARV at an MOI of 10 for 24 h. HFL-1 was included as a negative control. After collecting the extracellular supernatant, an equal volume of MEM was added to each well, and the cell membrane was destroyed by freezing and thawing three times. The supernatant was centrifuged at 13,000× *g* for 15 min at 4 °C to collect intracellular fraction, which was stored at −80 °C for virus titration. Virus titer was determined using an agar-covered plaque assay carried out in triplicate.

### 2.9. FRET-Based Senetically Encoded Indicators (Ateam)

Ateam was constructed by Dr. Imamura [18] and consisting of the ɛ subunit of the bacterial FoF1-ATP synthase, sandwiched with cyan-and yellow-fluorescent proteins. The detailed procedures were described previously [16,18].

### 2.10. Statistical Analysis

Data obtained from multiple independent experiments are expressed as mean ± standard errors (SE). The data were analyzed for statistical significance using The Duncan’s Multiple Range Test (MDRT) using Prism 8 software (GraphPad, San Diego, CA, USA). Means with common alphabet letter denotes no significance at *p* < 0.05. Each value represents the mean ± SE of three independent experiments.

## 3. Results

### 3.1. ARV Replication in Three Cancer Cell Lines

**Figure 1:**
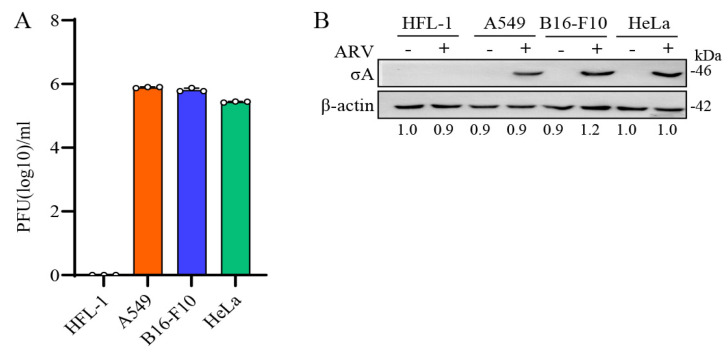
ARV replication in several cancer cell lines. (**A**) HFL-1, A549, B16-F10, and HeLa cell lines were infected with ARV at an MOI of 10 for 24 h. Plaque assay was performed to analyze the titer of ARV. HFL-1 cell as the negative control for this experiment. All data were obtained in three independent experiments, error bars indicate the mean ± SD. (**B**) Four cell lines were treated in the same way and examined using Western blots. Signals in all Western blots of β-actin were quantified with Image J.

Based on the high sensitivity of A549, B16-F10, and HeLa cancer cell lines to ARV, these three cancer cell lines were selected [5]. To confirm whether the ARV has good oncolytic activity and can replicate in these cancer cell lines, the structural protein σA was detected as evidence that the cells were infected by the virus. HFL-1, A549, B16-F10, and HeLa cell lines were infected with ARV at an MOI of 10 for 24 h. The results of the plaque assay showed that except HFL-1, the other cancer cells all had virus proliferation and virus plaque production (Figure 1A). In the Western blots, cell lysates were collected with 2.5× sample buffer dye. The results showed that σA protein was not detected in HFL-1 cells, while σA protein was detected in ARV-infected A549, B16-F10, and HeLa cancer cells (Figure 1B). Our results revealed that the σA protein could be detected in these cancer cell lines, and the same observation was also noticed in plaque assays. It means that ARV can infect these cancer cell lines, but not normal human lung cells, which proves that ARV does have the ability to target cancer cell lines. To further examine whether ARV induces cell fusion, known as a syncytium, all cancer cell lines were infected with ARV at an MOI of 10 for 24 h. Our results revealed that ARV can induce different degrees of cancer cell fusion. ARV-induced cytopathic effects (CPE) are shown in Supplementary Figure S1A. To test the replication of ARV in different cancer cell lines, all cancer cell lines were infected with different MOI (0.01 to 10) for different time points. Plaque assays were carried out to analyze the titers of ARV. Virus titers were obtained in these cancer cell lines in three independent experiments as shown in Supplementary Figure S1B. Collectively, ARV can infect these cancer cell lines, but not human normal lung cells, which proves that ARV does have the ability to target cancer cell lines.

### 3.2. ARV Infection and σA Transfection Increased c-myc, HIF-1α, and glut1 in Cancer Cell Lines

**Figure 2:**
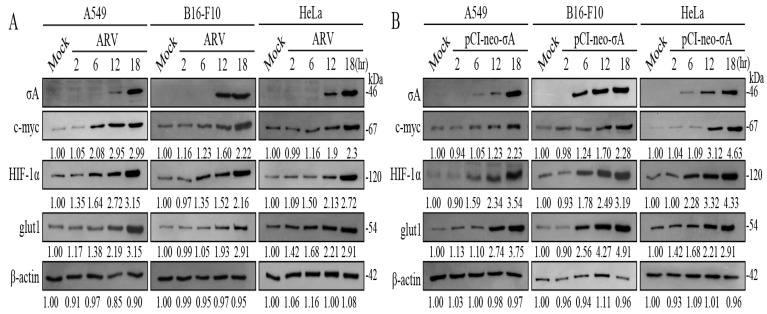
ARV infection and σA transfection increased of c-myc, HIF-1α, and glut1 in cancer cell lines. (**A**) A549, B16-F10, and HeLa cancer cell lines were infected with ARV at an MOI of 10. Cell lysates were collected with 2.5× sample buffer dye at 2, 6, 12, and 18 h post-infection for Western blots. (**B**) Cancer cell lines were transfected with the pCI-neo-σA plasmid at 2, 6, 12, and 18 h. Cell lysates were collected with 2.5× sample buffer dye and examined using Western blots. Signals in all Western blots were quantified with Image J and normalized to β-actin as shown in Supplementary Figure S2.

The c-myc and HIF-1α are important promoters of oncogenes in the carbohydrate metabolism of cancer cells. It can enable cancer cells to obtain more energy in an unfavorable growth environment to promote the growth of cancer cells [5]. To explore the effect of ARV on the regulation of the HIF-1α/c-myc/glut1 pathway in cancer cells, cancer cell lines were infected with ARV at an MOI of 10, and samples were collected in 2.5× sample buffer dye after infection 2, 6, 12 and 18 h for Western blotting. The results of Western blots showed that with the increase of σA protein expression, the expression of HIF-1α, c-myc, and glut1 proteins also increased, confirming that ARV can regulate HIF-1α/c-myc/glut1 pathway to further promote the glycolysis pathway (Figure 2A). Signals in all Western blot data in panel 2A were quantified using Image J software 1.53t (Supplementary Figure S2). It has been previously reported that ARV σA protein can promote the glycolysis pathway of Vero cells, and that σA protein also promotes HIF-1α [16]. It is the key to confirm whether σA protein affects HIF-1α and c-myc and may indirectly affect its downstream glut1 protein so that cancer cells can increase the absorption of glucose. Transfection was not toxic to cancer cell lines (Supplementary Figure S3). The cancer cell lines transfected with the pCI-neo-σA plasmid at 2, 6, 12, and 18 h, and cell lysates were collected with 2.5× sample buffer dye and examined using Western blots. The results of Western blots showed that the expression of c-myc, HIF-1α, and glut1 protein increased with the expression of σA protein (Figure 2B). In addition, ARV infection and σA shRNA transfection altered levels of c-myc, HIF-1α, and glut1 in cancer cell lines. Three cancer cell lines transfected with the σA shRNA for 18 h followed by ARV infection at an MOI of 10 for 24 h were examined using Western blots. It was found that the expression of c-myc, HIF-1α, and glut1 all had a downward trend, so it can be confirmed that σA protein can regulate the HIF-1α/c-myc/glut1 pathway (Supplementary Figure S4). Previous studies have demonstrated that transfection of the pcDNA3.1-p17 plasmid into Vero and HeLa cells can inhibit the mRNA transcription activity of c-myc by real-time PCR. The cancer cell lines transfected with the pcDNA3.1-p17 plasmid at 2, 6, 12, and 18 h were examined using Western blots. The results of Western blots showed that the c-myc protein of the three cancer cell lines decreased slightly with an increase in p17 protein expression, thus affecting the downstream glut1 protein, while HIF-1α protein was not affected (Supplementary Figure S5). It can be inferred that HIF-1α has no effect on the expression of p17 protein, while c-myc is regulated by p17 protein, which in turn affects the expression of glut1 protein downstream.

### 3.3. ARV σA Protein Upregulates c-myc, HIF-1α, and glut1 in mRNA Level

**Figure 3:**
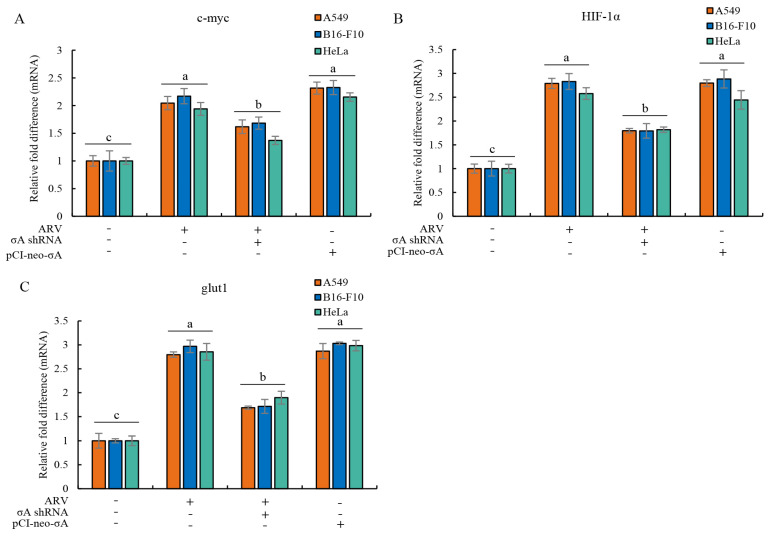
ARV infection and σA transfection upregulate mRNA levels of c-myc, HIF-1α, and glut1 in cancer cell lines. (**A**–**C**) cancer cell lines were infected with ARV or transfection the pCI-neo-σA or σA shRNA vectors for 6 h followed by infection with ARV at an MOI of 10 for 18 h. Cell lysates were collected for examining the mRNA levels of c-myc (**A**), HIF-1α (**B**), and glut1 (**C**) using quantitative real-time RT-PCR. Each value represents mean± SE from three independent experiments, determined using Duncan’s Multiple Range Test. Similar alphabets (a, b, c) denote no significance at *p* < 0.05.

Based on the above experimental results, it can be found that the ARV σA protein promotes the expression of c-myc, HIF-1α, and glut1 proteins at the protein level (Figure 2). It was confirmed that σA protein can enter the nucleus for regulation [17], indicating that σA may regulate and affect c-myc, HIF-1α, and glut1 proteins at the gene level. Therefore, cancer cell lines were infected with ARV or transfected with either the pCI-neo-σA or σA shRNA vectors, respectively. After transfection for 6h followed by infection with ARV at an MOI of 10 for 18 h, cell lysates were collected for examination of the mRNA levels of the three target proteins quantitative real-time RT-PCR. The results show that both ARV infection and pCI-neo-σA plasmid transfection can increase the mRNA levels of the three target proteins by about 2–3 times, confirming that ARV can regulate the gene transcription of the three proteins (Figure 3). Combined with the above experimental results, it is confirmed that σA protein can regulate these target proteins. The expressed σA protein could be detected in the cancer cell lines using Western blotting (Supplementary Figure S6).

### 3.4. Inhibition of c-myc or HIF-1α and Overexpression of σA Reduce the Virus Yield in Cancer Cell Lines

**Figure 4:**
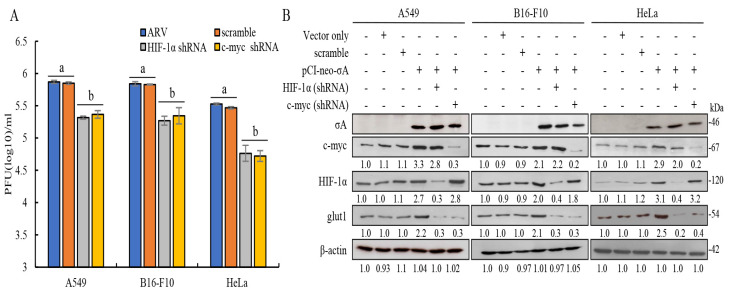
Knockdown of c-myc or HIF-1α by shRNAs reduced the expression levels of glut1 and virus yields in these cancer cell lines. (**A**)Three cancer cell lines were transfected with c-myc or HIF-1α shRNAs for the first 6 h, later infected with ARV at an MOI of 10 for 24 h. Plaque assay was performed to analyze virus titers. Each value represents mean± SE from three independent experiments, determined using Duncan’s Multiple Range Test. Similar alphabets (a, b) denote no significance at *p* < 0.05. (**B**) Three cancer cell lines co-transfected with the pCI-neo-σA and c-myc or HIF-1α shRNAs for 24 h. Cell lysates were collected and examined using Western blots. Densitometry analysis results for Western blot are expressed as percentages representing levels of c-myc, HIF-1α, and glut1 normalized to β-actin.

To understand whether σA protein regulates the expression of glut1 downstream through c-myc and HIF-1α to promote cancer cells to obtain more ATP, we inhibited the expression of c-myc and HIF-1α with shRNAs; subsequent to this, the effects of c-myc and HIF-1α on the expression of downstream glut1 protein were analyzed. The cancer cell lines were transfected with c-myc or HIF-1α shRNAs for the first 6 h and later infected with ARV at an MOI of 10 for 24 h. Plaque assay results showed that knockdown of c-myc and HIF-1α by shRNAs reduced virus yields (Figure 4A). It can be directly confirmed that c-myc and HIF-1α can affect the replication of ARV in these cancer lines. The effect of inhibiting the expression of c-myc and HIF-1α on the downstream protein glut1 was examined using Western blotting. The results confirmed that knockdown of c-myc or HIF-1α by shRNAs for 24 h led to the expression of the downstream protein glut1 being greatly reduced (Figure 4B), but c-myc and HIF-1α did not affect each other’s protein expression. Signals in Western blot in panel 4B were quantitated using Image J software (Supplementary Figure S7).

### 3.5. Visualization of Adenosine Triphosphate (ATP) Levels Inside Cells Infected with ARV in Cancer Cell Lines

**Figure 5:**
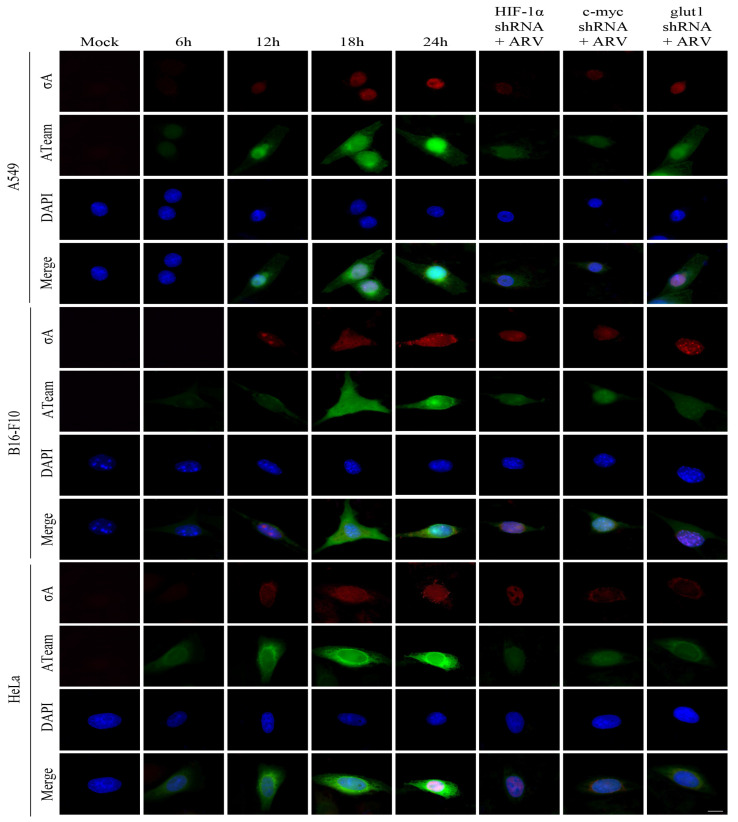
Visualization of adenosine triphosphate (ATP) levels inside cancer cell lines infected with ARV. To measure intracellular ATP levels, the cancer cell lines were transfected with the ATeams with or without c-myc, HIF 1α, and glut1 shRNAs for 18 h followed infection with ARV at different time points and MOI of 10 as indicated. The cells were observed and photographed with a fluorescence microscope (scale bar, 20 μm). The fluorescence density was quantitated using Image J software as shown in Supplementary Figure S8A.

**Figure 6:**
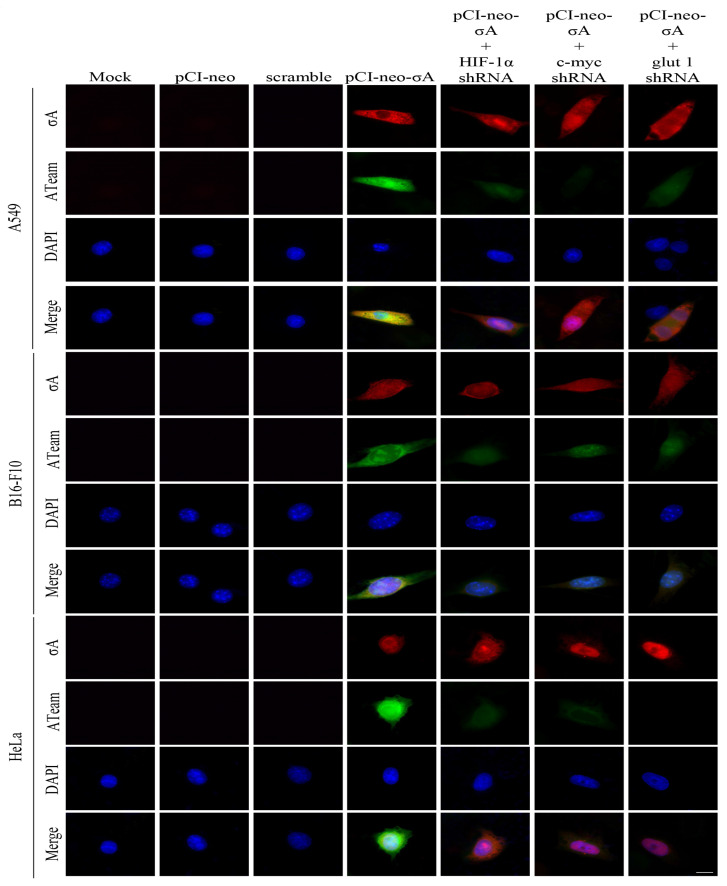
Visualization of adenosine triphosphate (ATP) levels inside cancer cell lines transfected with the pCI-neo-σA plasmid. To measure intracellular ATP levels, A549, B16-F10, and HeLa cancer cell lines were co-transfected with ATeams and the pCI-neo-σA plasmid with or without c-myc, HIF 1α, and glut1 shRNAs for 24 h. The cells were observed and photographed with a fluorescence microscope (scale bar, 20 μm). The fluorescence density was quantitated using Image J software as shown in Supplementary Figure S8B.

Previous studies have shown that ARV σA protein can promote glycolysis-related enzymes in Vero cells, promote the TCA cycle and other energy metabolism-related pathways, and increase intracellular ATP in Vero cells [16]. We investigated in this study if similar phenomenon occurs in the cancer cell lines. To measure intracellular ATP levels, A549, B16-f10, and HeLa cancer cell lines were transfected with the ATeams with or without c-myc, HIF 1α, and glut1 shRNAs for 18 h followed by ARV infection at different time points and MOI of 10 as indicated. The green fluorescence emitted by the Ateams represents the level of ATP energy. The stronger the green fluorescence, the more ATP in the cell, and vice versa. It was confirmed by the fluorescence results that if cancer cell lines were infected with ARV, the synthesis of ATP could be promoted. As the expression of σA protein increased, the green fluorescence also increased (Figure 5). If the expression of c-myc, HIF-1α, and glut1 are inhibited, the intensity of green fluorescence is greatly reduced. To confirm that σA protein can directly regulate the HIF-1α/c-myc/glut1 signaling pathway, the pCI-neo-σA and shRNA vectors were co-transfected to inhibit the expression of c-myc, HIF 1α, and glut1, and to observe whether it affects the expression of cell energy generation. According to the fluorescent results of the transfected cancer cell lines, the results were similar to the above (Figure 6). The data shown in Figure 5 and Figure 6 were quantitated by fluorescence density analysis using Image J software (Supplementary Figure S8A,B). Our results demonstrated that the ARV σA protein can regulate the energy metabolism of the cells through the HIF-1α/c-myc/glut1 signaling pathway in these cancer cell lines, and all three target proteins can affect the production of ATP in the cells.

### 3.6. ARV Infection and pCI-neo-σA Transfection Alter Levels of LDHA, PKM2, OGDH, and Gls in Cancer Cell Lines

**Figure 7:**
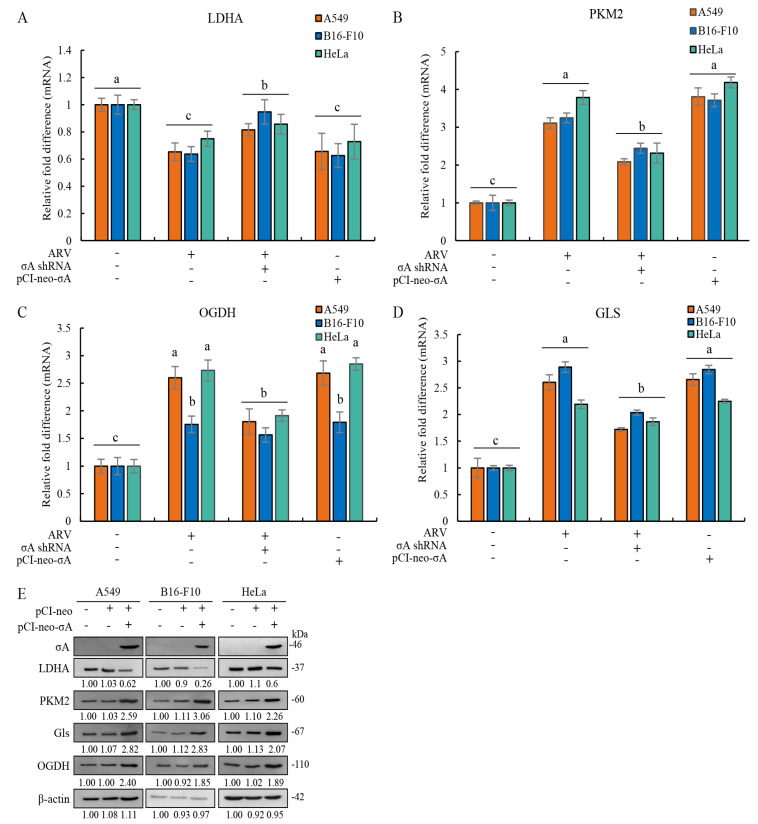
ARV infection and ARV σA transfection alter levels of LDHA, PKM2, OGDH, and Gls in A549, B16-F10, and HeLa cancer cell lines. (**A**–**D**) The cancer cell lines were transfected with or without the pCI-neo-σA or σA shRNA vectors for 6 h followed infection with ARV at an MOI of 10 for 18 h. Cell lysates were collected for examining the mRNA levels of LDHA (**A**), PKM2 (**B**), OGDH (**C**), and Gls (**D**) using quantitative real-time RT-PCR. Each value represents mean ± SE from three independent experiments, determined using Duncan’s Multiple Range Test. Similar alphabets (a, b, c). (**E**) Cancer cell lines were transfected with the pCI-neo-σA plasmid for 24 h followed by Western blot assay. Signals in all Western blots were quantified with Image J and normalized to β-actin as shown in Supplementary Figure S9. In this study, all uncropped blots are shown in Supplementary Figure S10.

In this study, we also explored whether σA protein can regulate the lactic acid fermentation and glutamine metabolism pathways. A previous report suggested that HIF-1α and c-myc in cancer cells can regulate the lactic acid fermentation pathway and the glutamine metabolism pathway [5]. Our results showed that σA protein reduces the mRNA level of lactic acid fermentation pathway enzyme LDHA and increases the mRNA level of PKM2 that catalyzes the last step within glycolysis (Figure 7A,B), indicating that it can promote the entry of pyruvate used in the TCA cycle (Figure 7B). In the glutamine metabolic pathway, glutaminase is labeled as a protein marker for detection. The experimental results showed that the mRNA level of Gls has an upward trend (Figure 7C). A similar trend was also found in OGDH (Figure 7D). According to the results, it can be confirmed that the σA protein can inhibit the lactic acid fermentation pathway and promote the glutamine metabolism pathway at the mRNA level to increase the TCA cycle. We next wanted to explore whether the results of real-time PCR are consistent with those of the Western blots. The cancer cell lines were transfected with ARV σA for 24 h and examined using Western blots. The results are the same as real-time PCR; LDHA was reduced while the expression of PKM2, Gls, and OGDH increased (Figure 7E). Signals in Western blots in panel E were quantified with Image J (Supplementary Figure S9). According to the above results, the σA protein may promote the entry of pyruvate into the TCA cycle, generating a large amount of energy, and promoting virus replication [16].

## 4. Discussion

Oncolytic virotherapy is an emerging therapy that uses viruses or re-edited viruses for cancer treatment [15]. In 1949, hepatitis B virus was first used to treat malignant lymphoma Hodgkin’s disease (HD) as a clinical trial, opening a new chapter in oncolytic therapy [19]. Previous studies have confirmed that ARV has good oncolytic activity, can induce cancer cell apoptosis, promote cancer cell immune response and expose tumor-associated antigens to the immune system [7,8,9,10]. The FDA issued a drug license for the isolated component of MRV Type 3 in 2015 and named Reolysin (currently renamed Pelareorep) for the treatment of glioma [11]. In 2017, the FDA also issued a license for the treatment of metastatic breast cancer. In recent years, many reports have confirmed that this drug has good anticancer activity against pancreatic ductal adenocarcinoma, metastatic colorectal cancer, and prostate cancer [12,20]. However, this virus is a direct use of non-genetically modified virus isolates as drugs to treat cancer. Since this virus can infect mammals and humans, there are potential factors that may cause the body to produce antibodies and immune rejection due to infection. Therefore, compared with the hidden dangers of MRV, ARV has more potential to be developed as an anticancer drug because of its non-animal characteristics. This work provides that ARV can infect cancer cells, but not human normal lung cells, which proves that ARV does have the ability to target cancer cells.

Viruses ensure the best environment for their replication and transmission by changing the metabolic pathway of the host cell. Many pieces of literature have demonstrated that after virus infection of host cells, it can regulate cellular glycolysis, fatty acid metabolism, and glutamine catabolism/glutaminolysis, the PI3K/Akt/mTOR pathway, c-myc, HIF-1α, and tumor formation [3,16,17,21]. The generation of a large amount of energy is conducive to the replication and spread of the virus. The literature shows that different viruses have different metabolic pathways that regulate host cells. In-depth research on regulatory mechanisms can help to find new directions for disease treatment by re-editing viruses and other methods [22,23]. One of the most frequently altered signaling pathways in cancer is the PI3K/Akt/mTOR pathway. Through post-translational regulation and induction of transcriptional programs, the PI3K/Akt/mTORC1 pathway coordinates the uptake and utilization of multiple nutrients, including glucose, glutamine, nucleotide, and lipid metabolism, in a manner optimal for cell growth to facilitate cancer cells proliferation and spread [24]. We have previously demonstrated that the p17 and σA proteins of ARV can inhibit the PI3K/Akt/mTORC1 pathway and promote the expression of PSMB6, thereby inducing cell autophagy and promoting virus replication [14,25,26]. The p17 protein has also been shown to inhibit cell growth, arrest the cell cycle and inhibit the growth of various cancer cells [8]. Another study also demonstrated that σA protein can promote the expression of GDH and IDH3B, thereby activating glycolysis, promoting the TCA cycle, and making host cells to generate more energy for the benefit of Viral replication [16].

To meet the needs of rapid proliferation and metastasis, cancer cells must produce a large amount of energy for cancer cells to use, so they have evolved different metabolic pathways from normal cells [5,27]. The life cycle of viruses relies on the host cell’s metabolic machinery to provide it with the energy necessary for proliferation. Previous studies have confirmed that many viruses provide the necessary energy by increasing the host’s ATP formation, such as increasing the intake and synthesis of nutrients such as sugars, proteins, and fats. These energies are used for viral genome synthesis, viral protein assembly, amino acid production, and virus budding [28]. In this study, it was confirmed that all three cancer cell lines could be infected by the ARV. The Ateam plasmid system was used to detect the amount of ATP produced in cancer cell lines during ARV infection and transfection of the p-CI-neo-σA plasmid [18]. The results confirmed that the three cancer cell lines could increase the intracellular ATP production when infected with ARV or transfected with the pCI-neo-σA plasmid. If the protein expression of c-myc, HIF-1α, and glut1 is inhibited, the synthesis of ATP in the cell will be reduced, so these three proteins are identified as the key proteins of energy production. This study is the first to demonstrate that ARV σA protein can promote ATP production in cancer cells to facilitate viral replication.

Viral infection confers new metabolic signatures on host cells that closely resemble metabolic changes in cancer cells, both exhibiting the Warburg effect. The Warburg effect means that compared with normal cells, cancer cells synthesize 70 times more lactic acid than normal cells during the metabolic process of consuming glucose. It has been shown that cancer cells prefer the lactic acid fermentation pathway for rapid energy gain. This metabolic pathway can occur in a hypoxic environment without the involvement of mitochondria and is therefore observed in abundance in most cancer cells [6,29,30]. Promoting nutrient consumption and anabolism in cancer cells can support viral replication or provide rapid cancer cell growth, respectively. Our findings reveal that the levels of mRNA and protein of LDHA, an important enzyme for lactic acid fermentation, tended to decrease after ARV-infected cancer cell lines. However, in the glutamine metabolism pathway, the important metabolic enzyme glutaminase and OGDH both have a significant upward trend in ARV-infected cancer cell lines, and the overexpression of σA protein in cancer cells has the same trend. After infecting host cells, it can suppress the lactic acid fermentation and enhance the glutamine metabolism pathway, thereby increasing the production of cellular ATP. Our previous report has demonstrated that ARV can alter the metabolic pathways of Vero and DF-1 cell lines that favor the viral life cycle [16]. DNA and RNA viruses have been shown to reprogram various aspects of host energy metabolism, including increased synthesis of the lactic acid fermentation pathway and pentose phosphate activity to support nucleotide, amino acid, and lipid synthesis [28]. For example, the Vaccinia virus can regulate lipid metabolism pathways in host cells. This virus can promote the entry of palmitate, the raw material for synthetic lipids, into mitochondria, helping host cells to synthesize lipids for use by viruses [31]. In addition, poxviruses have been shown to induce and depend on changes in host cell metabolism. Poxvirus C16 protein promotes HIF-1α by binding to a prolyl hydroxylase domain-containing protein (PHD2) to stabilize HIF-1α [32]. Our research confirmed that ARV σA promotes PSMB6 and inhibits Akt, which inhibits downstream SREBP1, ACC1 (acetyl-CoA carboxylase 1), and ACC2 (acetyl-CoA carboxylase 2), thereby inhibiting fatty acid synthesis and enhancing fatty acid oxidation to produce more energy for virus replication [17].

This study has demonstrated for the first time that ARV σA protein positively regulates the expression of c-myc and HIF-1α to promote the expression of glut1, thereby increasing the uptake of glucose by cells. After infection with the dengue virus, it can upregulate mRNA and protein levels of glut1 and hexokinase 2, which can increase the uptake of glucose to promote the TCA cycle and synthetic lipids [33]. In addition, certain viruses can induce the lactic acid fermentation pathway to generate energy in a low-oxygen environment, similar to tumor cells. For example, Herpes simplex virus 1 and 2 can promote glucose consumption and increase lactic acid synthesis to generate energy for virus use [34]. It has also been reported that c-myc and HIF-1α cooperate to regulate glycolysis and stimulate the Warburg effect [5]. In this work, we have found that ARV downregulates LDHA, the important enzyme of lactic acid fermentation. This may cause pyruvate to enter the mitochondria to promote cancer cells to the glycolysis pathway and affect the TCA cycle, to obtain more energy than lactic acid fermentation. Furthermore, the expression levels of glutaminase and glutamine dehydrogenase were also increased in cancer cell lines after ARV infection. We found that ARV σA protein was able to promote the metabolic pathway of glutamic acid, the enzyme glutaminase and GDH [16] that converts glutamic acid into α-ketoglutarate, an intermediate product of the TCA cycle. We have previously demonstrated that glutamine deficiency reduces viral protein synthesis of ARV with reduced expression of HIF-1α and that σA protein regulation of glutamine breakdown was essential in the life cycle of ARV [16]. These findings are consistent with a previous study demonstrating that HIF-1α can promote the expression of glutaminase, promote the conversion of glutamine into α-ketoglutarate, supplement the TCA cycle, and increase energy production [35]. In human cytomegalovirus, virus-infected human fibroblasts can increase glucose consumption, increase lactate production [36], regulate the fat synthesis pathway [37], and promote glutamine breakdown [35].

**Figure 8:**
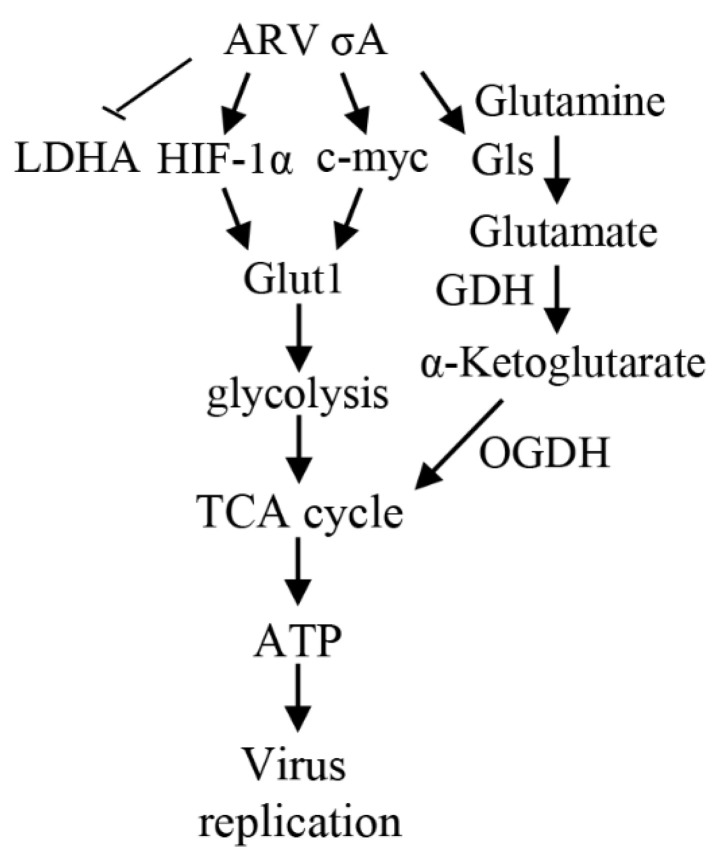
A model depicting that ARV σA enhances glycolysis and glutaminolysis to produce energy via the HIF-1α/c-myc/glut1 pathway to benefit ARV replication in A549, B16-F10, and HeLa cancer cell lines. →: activation; ⊥: inhibition. GDH: glutamate dehydrogenase [16].

This study provides a novel insight into the mechanisms underlying how ARV σA protein regulates metabolic pathway in cancer cell lines. ARV σA protein suppresses the lactic acid fermentation pathway preferred by cancer cells, allowing pyruvate to enter the TCA cycle and glucose to enter the mitochondria to produce more energy for virus replication. At the same time, it promotes the decomposition of glutamine, converts glutamine into α-ketoglutarate, and supplements the TCA cycle. These findings are consistent with a previous report suggesting that σA-modulated suppression of LDHA and activation of IDH3B and GDH to activate the mTORC1/eIF4E/HIF-1α pathways to upregulate glycolysis and the TCA cycle for virus replication in Vero cells [16]. All of the above pathways can prompt host cells to produce more energy for virus replication, and at the same time compete with cancer cells for the substances and energy needed for growth, thereby achieving an anti-cancer purpose. A model showing that ARV σA regulates glycolysis and glutaminolysis to produce more energy via the HIF-1α/c-myc/glut1 pathway to benefit ARV replication in cancer cell lines is shown in Figure 8.

## Figures and Tables

**Table 1:** Primers used in this study.

Gene	Sequences (5′–3′)	Expected Size (bp)
HIF-1α	F: ATGAAGTGTACCCTAACTAGCCG R: GCTTGAGTTTCAACCCAGACATA	452
c-myc	F: ATGCCCCTCAACGTGAACTTC R: GTCGCAGATGAAATAGGGCTG	183
glut1	F: ACTGGGCAAGTCCTTTGAGAT R: GTCCTTGTTGCCCATGATGGA	213
β-actin	F: TTAAGGAGAAGCTGTGCTACG R: GTTGAAGGTAGTTTCGTGGAT	208
LDHA	F: GCCCTCAGGAGGCTATACTT R: GCAAGTTCATCTGCCAAGTCC	225
PKM2	F: CCCGATCTGTGGAGATGCTG R: CGGATCTCAGGTCCCTTTGT	196
OGDH	F: GCTAGTCTCTTCCTTGACTG R: AACTTACTCATGCCATTGTC	184
Gls	F: GCTGTGTTCCATTGAGGTGAC R: ACTCGCTCGCCTGTGATTG	93

## Data Availability

Not applicable.

## Supplementary Materials

The following supporting information can be downloaded at: https://www.mdpi.com/article/10.3390/v15020523/s1. Figure S1: Oncolytic ARV replication in cancer cell lines HeLa, A549, and B16-F10; Figure S2: Quantification of Western blot results; Figure S3: Cell viability in three cancer cell lines; Figure S4: ARV infection and σA shRNA transfection alter levels of c-myc, HIF-1α, and glut1 in three cancer cell lines; Figure S5: ARV p17 downregulates the levels of c-myc, HIF-1α, and glut1 in three cancer cell lines; Figure S6: Confirmation of the expression levels of ARV σA protein; Figure S7: Quantification of signals in Western blots; Figure S8: Quantification of ATeams by fluorescence density analysis; Figure S9: Quantification of signals in Western blots; Figure S10: All original uncropped blots.
