# Supplementary material for: Oncolytic Avian Reovirus σA-Modulated Upregulation of the HIF-1α/C-myc/glut1 Pathway to Produce More Energy in Different Cancer Cell Lines Benefiting Virus Replication
Source: Viruses. 2023 Feb 13;15(2):523. doi: 10.3390/v15020523 (PMC9961784; doi:10.3390/v15020523)

Supplementary materials

Oncolytic avian reovirus σA-modulated upregulation of the HIF-1α/c-myc/glut1 pathway to produce more energy in different cancer cell lines benefiting virus replication

Chao-Yu Hsu[1, 2,†], Jing-Wen Huang[3,4, †], Wei-Ru Huang[3, 4], I-Chun Chen[2, 5], Ming-Shan Chen[6], Tsai-Ling Liao[2, 7], Yu-Kang Chang[8, 9], Muhammad Munir[10], Hung-Jen Liu[2, 3, 4 ,11, 12*]

[1]Division of urology, Department of Surgery, Tungs' Taichung MetroHarbor Hospital, Taichung 435, Taiwan

[2]Ph.D Program in Translational Medicine, National Chung Hsing University, Taichung 402, Taiwan

[3]Institute of Molecular Biology, National Chung Hsing University, Taichung 402, Taiwan

[4]The iEGG and Animal Biotechnology Center, National Chung Hsing University, Taichung 402, Taiwan

[5]Department of Psychiatry, Taichung Veterans General Hospital, Taichung 407, Taiwan

[6]Department of Anesthesiology, Ditmanson Medical Foundation Chia-Yi Christian Hospital, Chia-Yi 600, Taiwan

[7]Department of Medical Research, Taichung Veterans General Hospital, Taichung 407, Taiwan

[8]Department of Medical Research, Tungs' Taichung MetroHarbor Hospital, Taichung 435, Taiwan

[9]Department of Post-Baccalaureate Medicine, National Chung Hsing University, Taichung 402, Taiwan

[10]Department of Biomedical and Life Sciences, Lancaster University, Lancashire LA1 4YW, UK

[11]Rong Hsing Research Center for Translational Medicine, National Chung Hsing University, Taichung 402, Taiwan

[12]Department of Life Sciences, National Chung Hsing University, Taichung 402, Taiwan

*Correspondence: hjliu5257@nchu.edu.tw; Tel.: +886-4228-40485 (ext. 243); Fax: +886-4228-74879

†These authors contributed equally to this work.

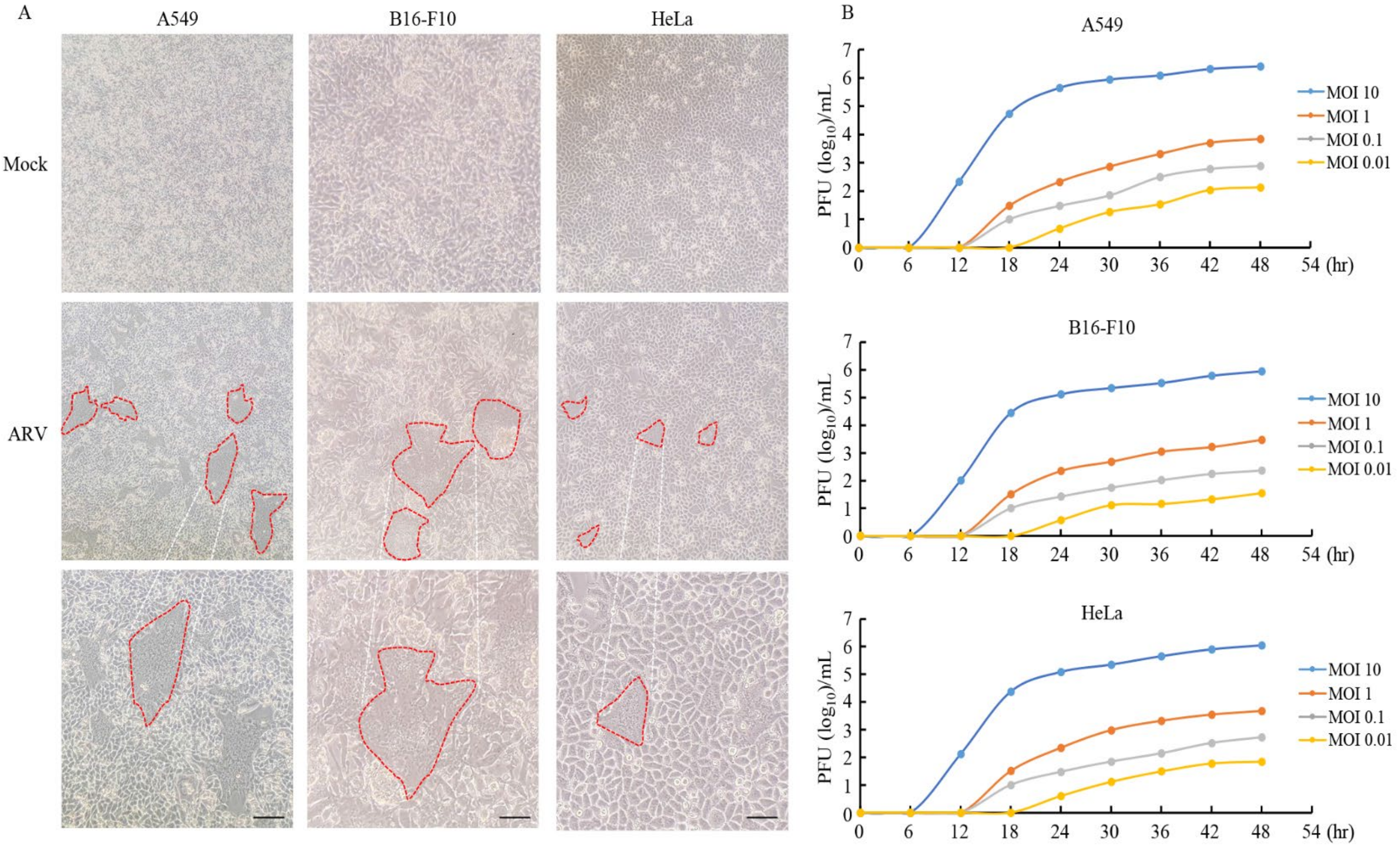

Supplementary Figure S1. Oncolytic ARV replication in cancer cell lines HeLa, A549, and B16-F10. (A) All cancer cell lines were infected with ARV at an MOI of 10 for 24 hours. The representative regions of cell fusion, known as a syncytium, induced by oncolytic ARV were indicated by the red dotted frame. Enlarged images correspond to the region indicated by the red dotted frame in the low panels. Scale bar, 100 μm. **(B)** All cancer cell lines were infected with ARV at the indicated MOI for the respective time points. Plaque assay was performed to analyze the titers of ARV. All data were obtained in three independent experiments, error bars indicate the mean± SE.

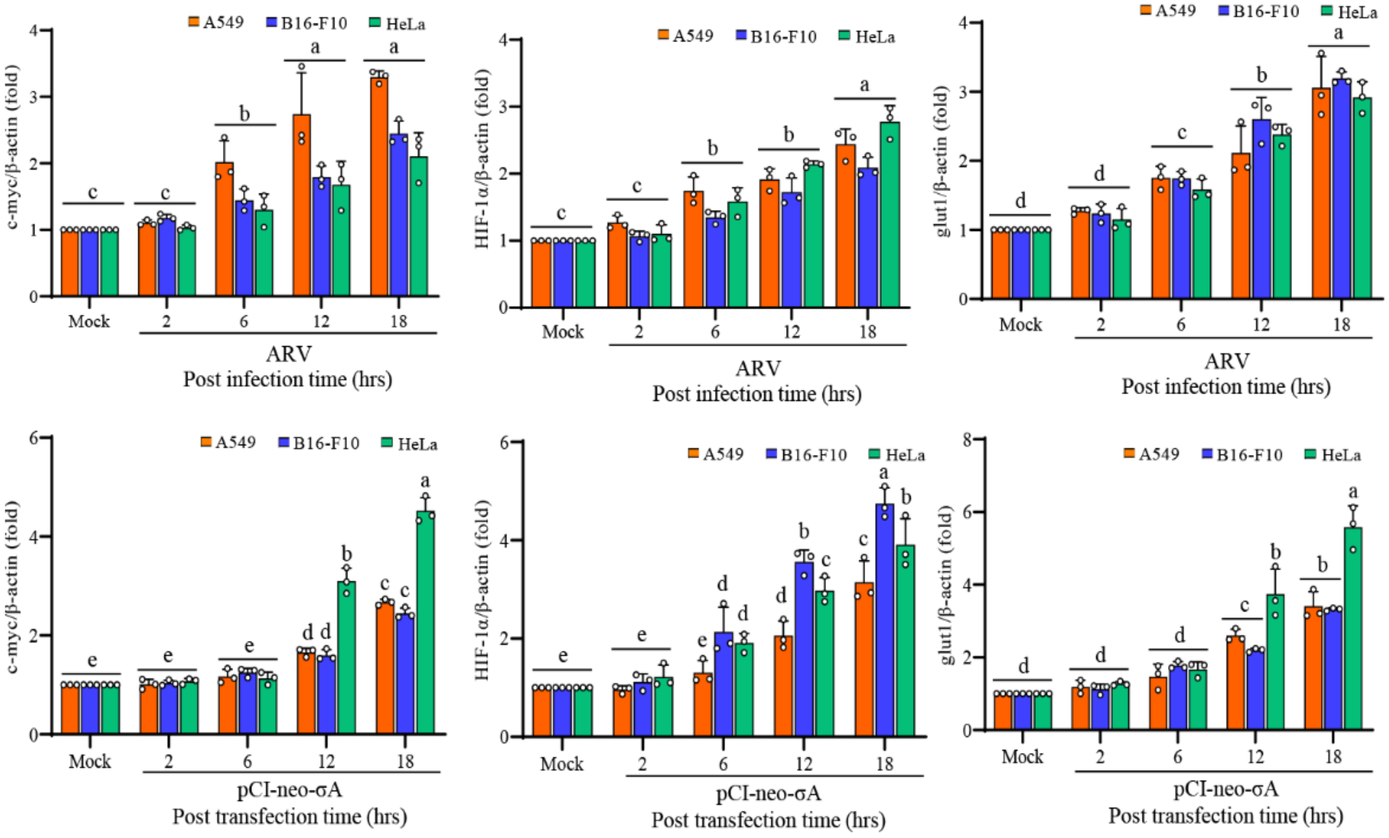

Supplementary Figure S2. Quantification of Western blot results. Signals in all Western blots in Fig. 2 were quantified using Image J software and normalized to mock-treated β-actin. Values for mock-treated cells were considered 1-fold. Each value represents mean± SE from three independent experiments, determined by Duncan's Multiple Range Test. Similar alphabets (a, b, c) denote no significance at p<0.05.

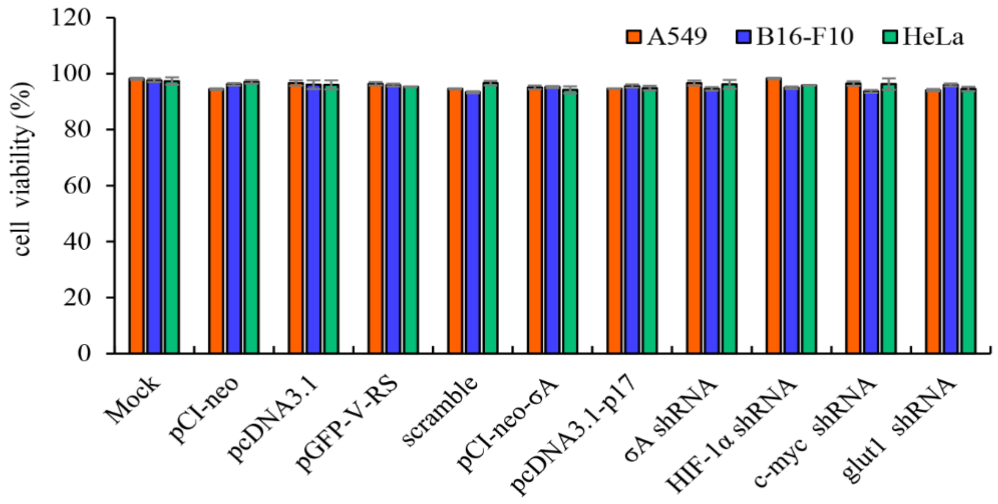

Supplementary Figure S3. Cell viability in three cancer cell lines. The cancer cell lines (A549, B16-F10, and HeLa) transfected with the indicated plasmids for 24 hours. Cell viability was examined by MTT assay. Similar results were obtained in three independent experiments.

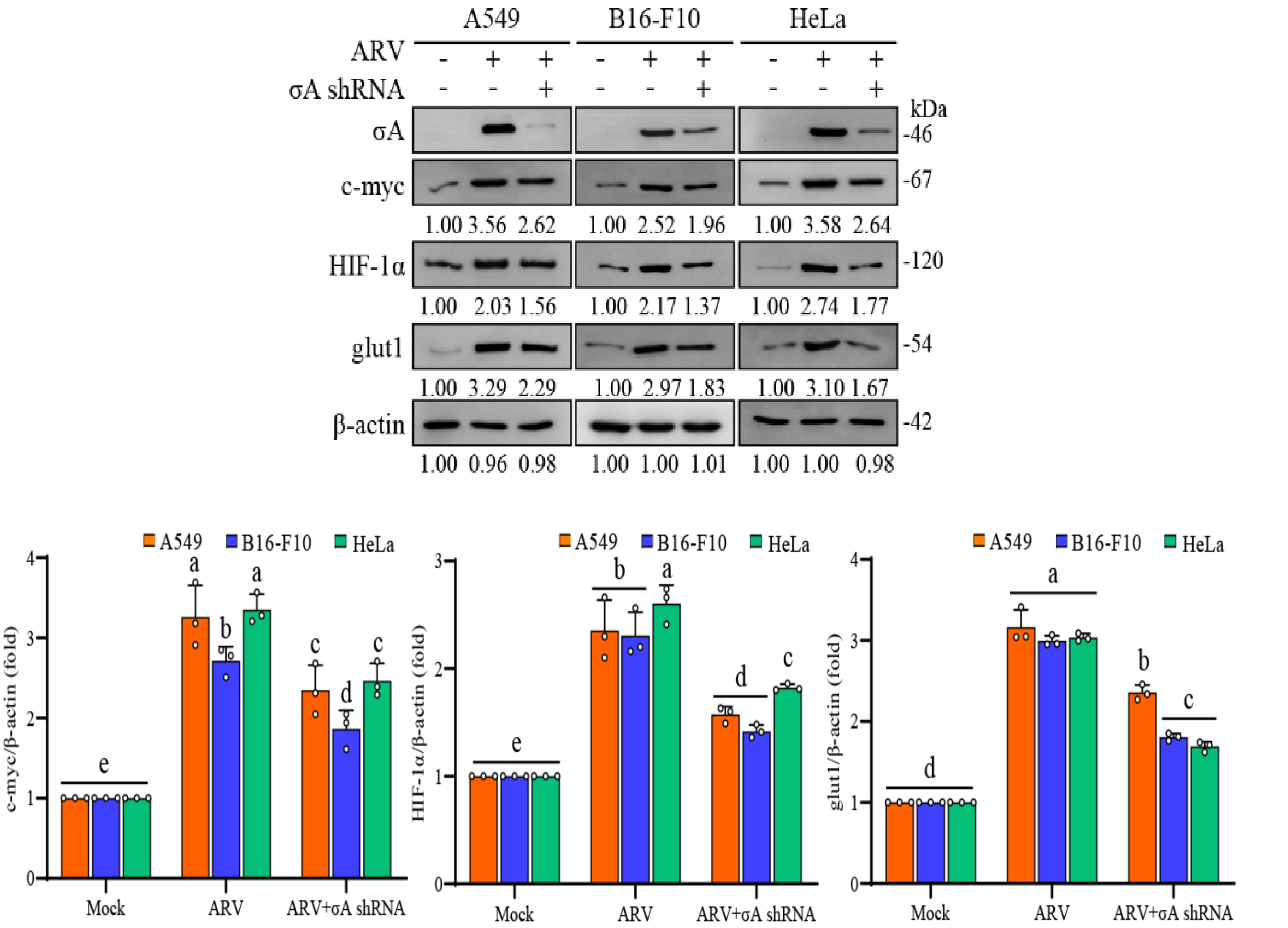

Supplementary Figure S4. ARV infection and σA shRNA transfection alter levels of c-myc, HIF-1α, and glut1 in three cancer cell lines. Three cancer cell lines (A549, B16-F10, and HeLa) were transfected with the σA shRNA for 18 hours followed by ARV infection at an MOI of 10 for 24 hours and examined by Western blots. Signals in all Western blots in the upper panel were quantified with Image J and normalized to β-actin. Each value represents mean± SE from three independent experiments, determined by Duncan's Multiple Range Test. Similar alphabets (a, b, c) denote no significance at $p<0.05$.

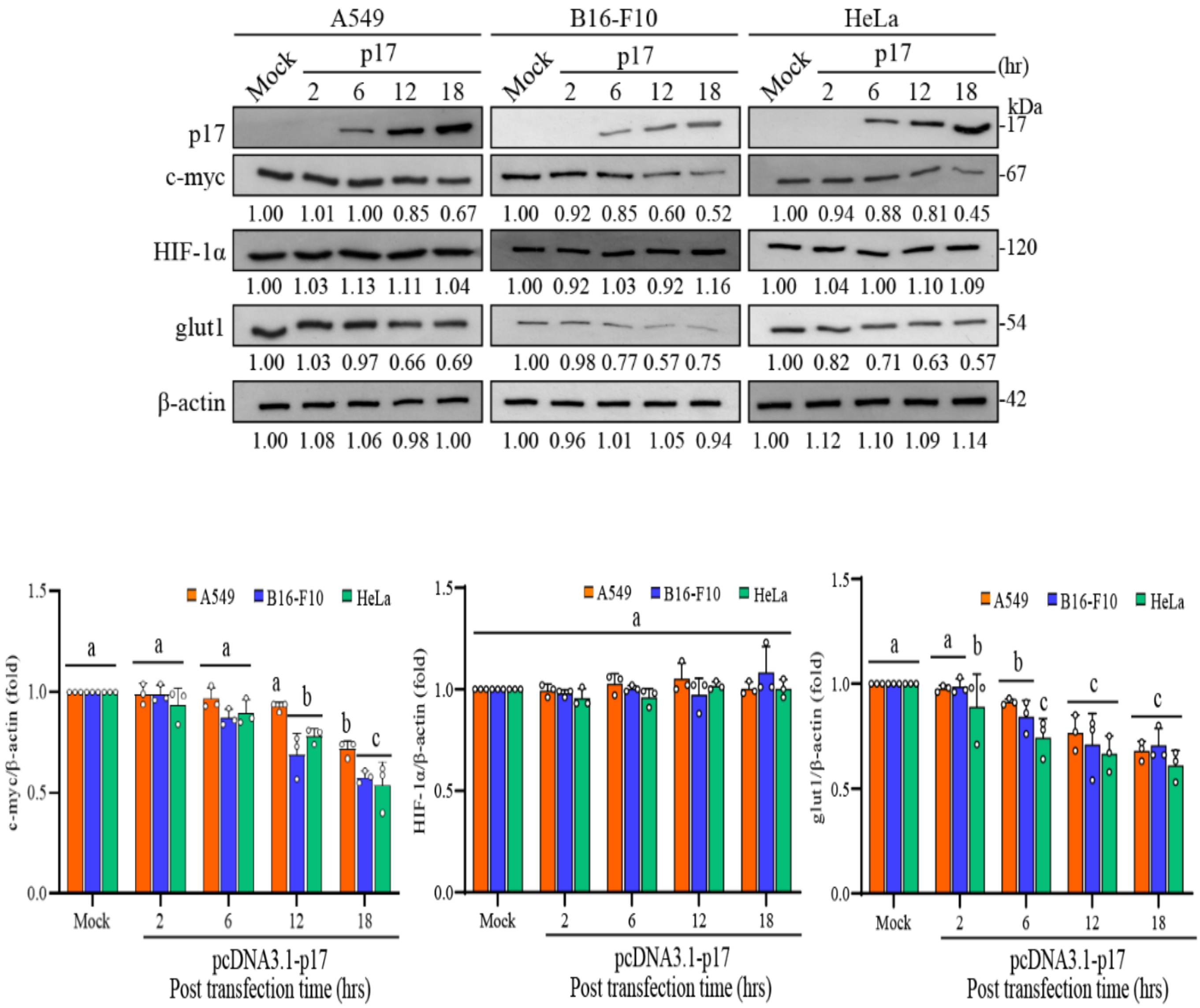

Supplementary Figure S5. ARV p17 downregulates the levels of c-myc, HIF-1α, and glut1 in three cancer cell lines. Three cancer cell lines transfected with the pcDNA3.1-p17 plasmid at 2, 6, 12, and 18 hours were collected and examined by Western blots. Signals in all Western blots in the upper panel were quantified with Image J and normalized to β-actin. Each value represents mean± SE from three independent experiments, determined by Duncan's Multiple Range Test. Similar alphabets (a, b, c) denote no significance at $p<0.05$.

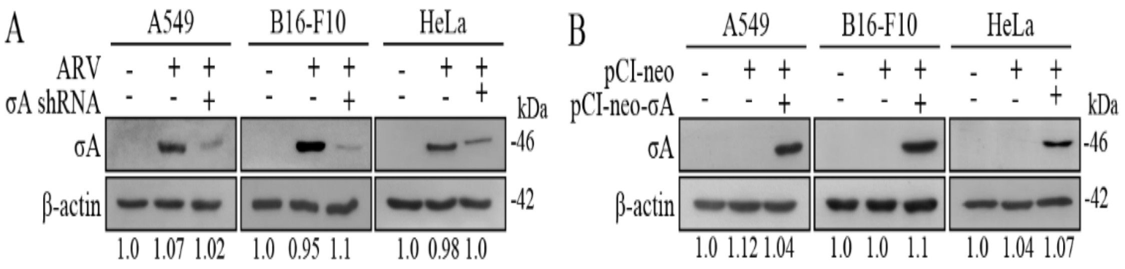

Supplementary Figure S6. Confirmation of the expression levels of ARV σA protein. (**A**)Three cancer cell lines were transfected with or without σA shRNA for 18 hours followed by ARV infection at an MOI of 10 for 24 hours were collected and examined by Western blots. (**B**) The cancer lines transfected with or without the pCI-neo-σA plasmid for 24 hours were examine by Western blots. Signals in all Western blots were quantified with Image J and normalized toβ- actin.

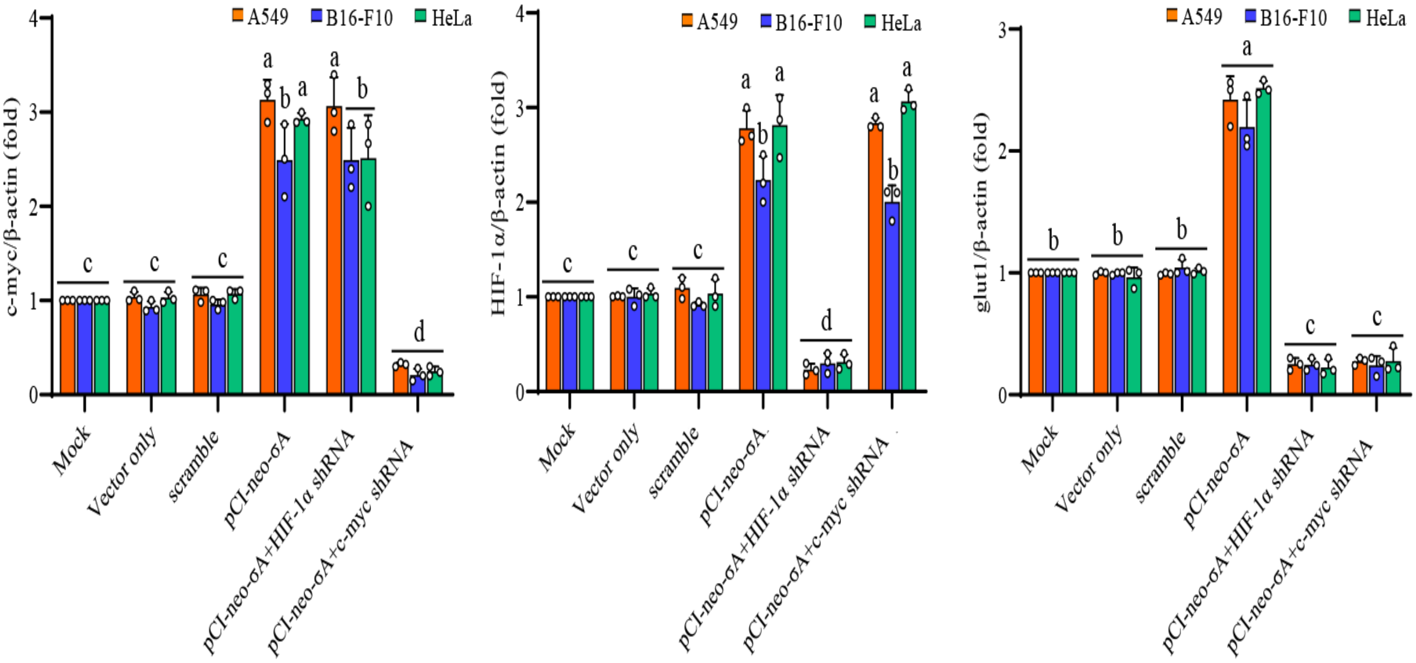

Supplementary Figure S7. Quantification of signals in Western blots. Signals in Western blots in Fig. 4 were quantitated using Image J software and normalized to mock-treated β-actin. Values for mock-treated cells were considered 1-fold. Each value represents mean± SE from three independent experiments, determined by Duncan's Multiple Range Test. Similar alphabets (a, b, c) denote no significance at $p<0.05$.

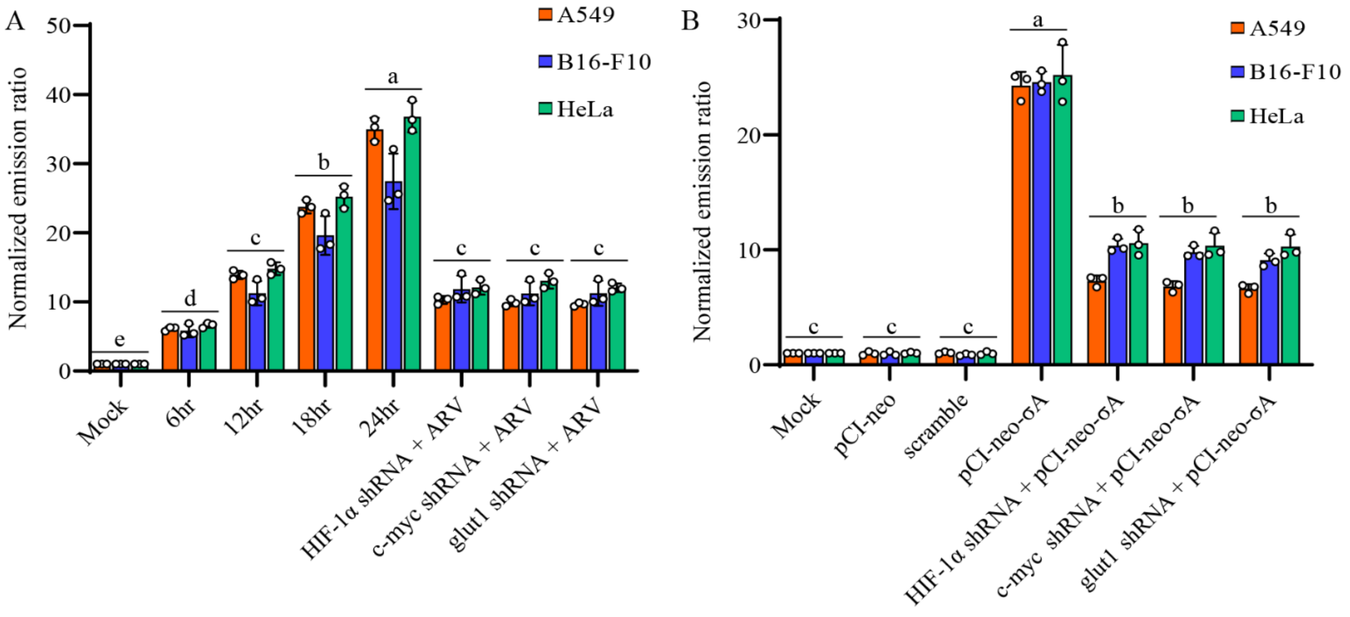

Supplementary Figure S8. Quantification of ATeams by fluorescence density analysis. (**A**)The data of Fig. 5 was quantitated by using Image J software and normalized to mock-treated ATeams. Each value represents mean± SE from three independent experiments, determined by Duncan's Multiple Range Test. Similar alphabets (a, b, c) denote no significance at $p<0.05$. (**B**) The data of Fig. 6 was quantitated by fluorescence density analysis using Image J software and normalized to mock-treated ATeams. Each value represents mean± SE from three independent experiments, determined by Duncan's Multiple Range Test. Similar alphabets (a, b, c) denote no significance at $p<0.05$.

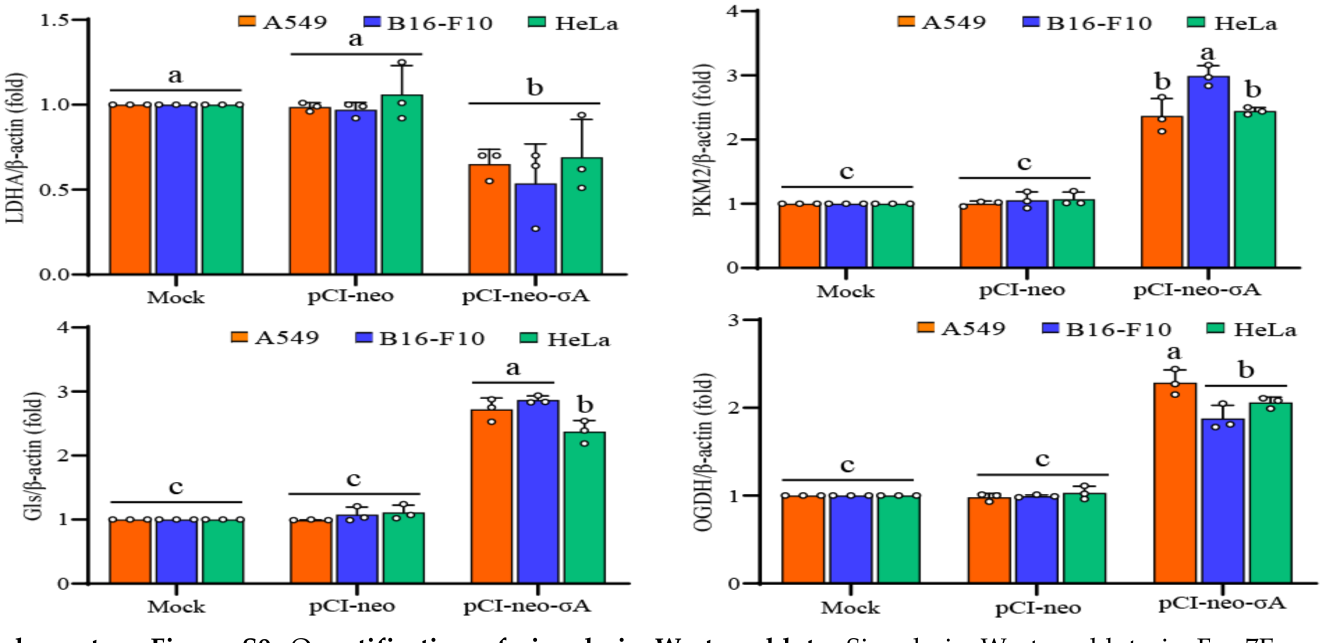

Supplementary Figure S9. **Quantification of signals in Western blots.** Signals in Western blots in Fg. 7E were quantitated using Image J software and normalized to mock-treated actin. Values for mock-treated cells were considered 1-fold. Each value represents mean± SE from three independent experiments, determined by Duncan's Multiple Range Test. Similar alphabets (a, b, c) denote no significance at $p<0.05$.

Supplementary Figure S10: All original uncropped blots.

Fig. 1A

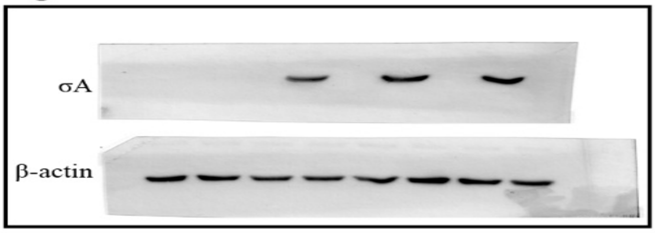

Fig. 2A

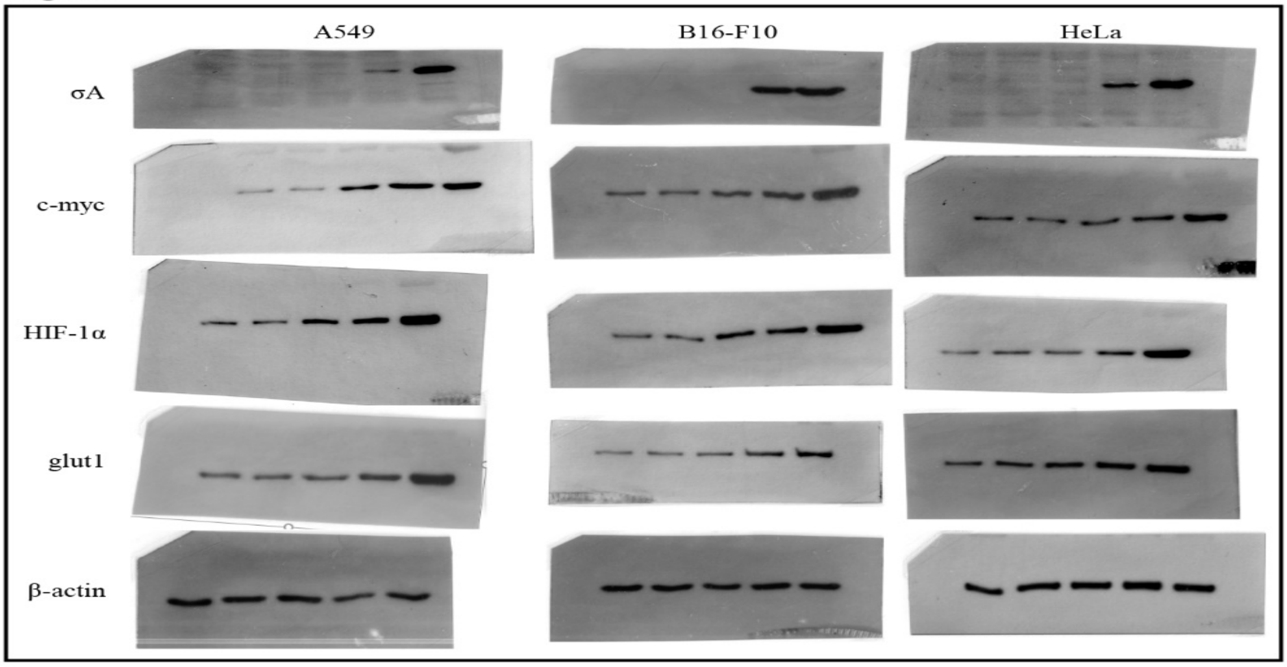

Fig. 2B

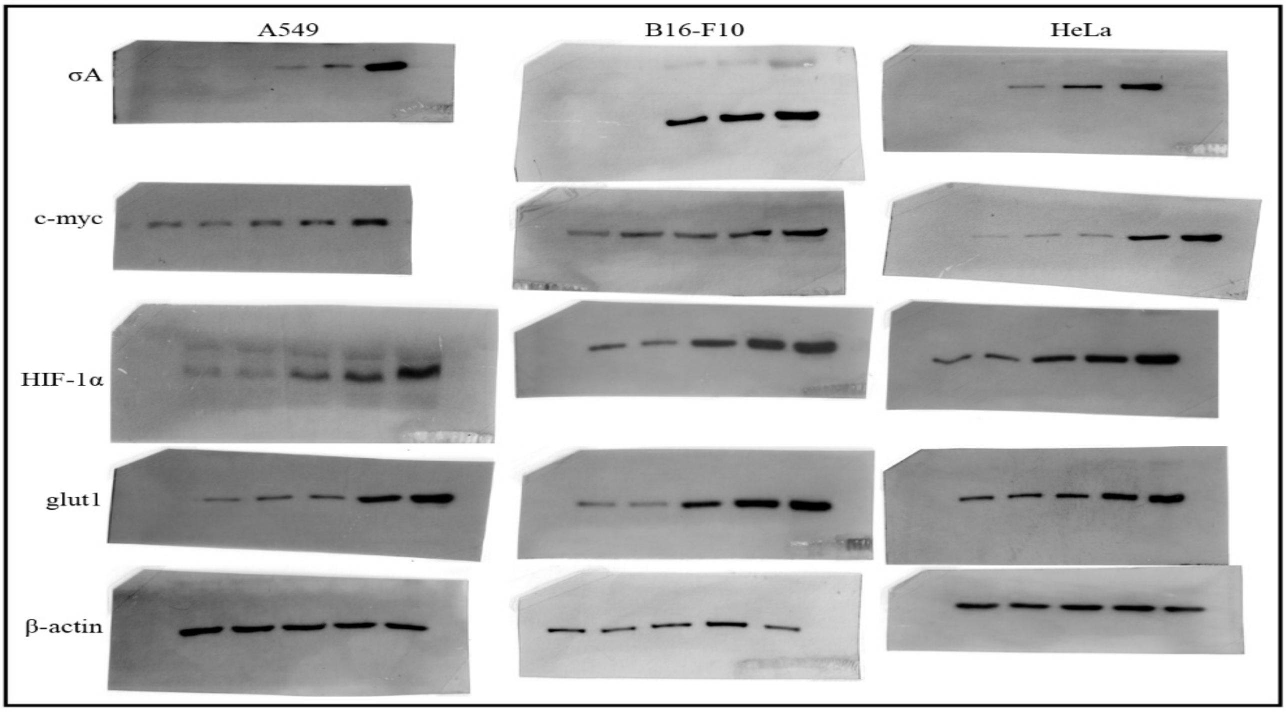

Fig. 4A

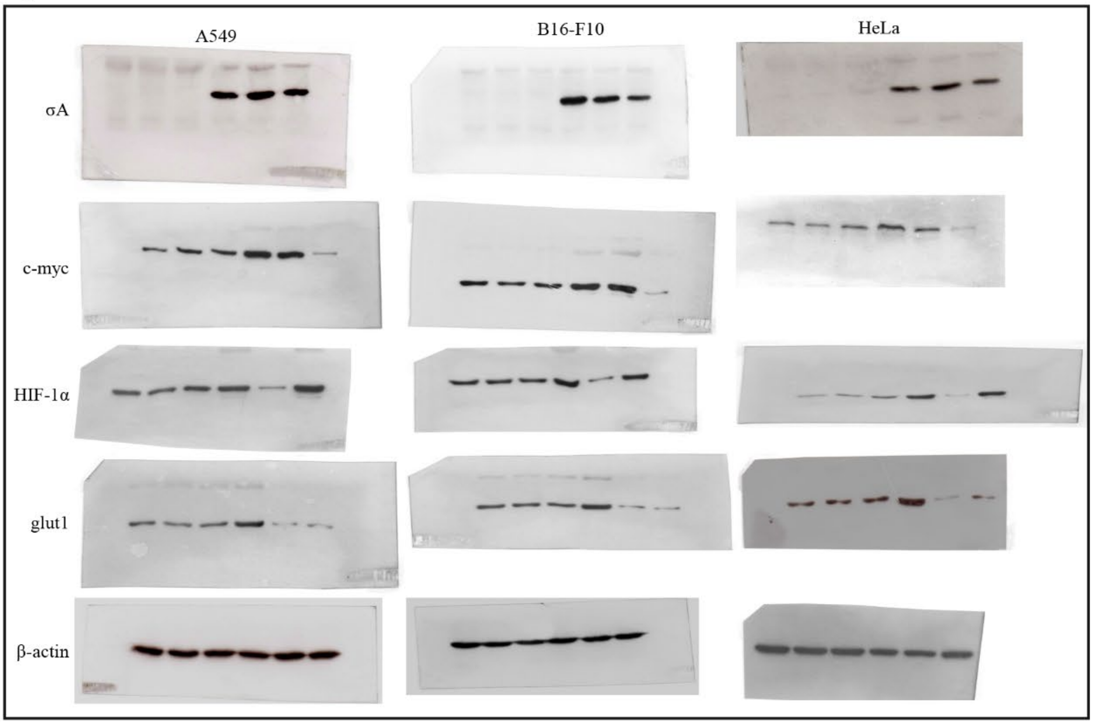

Fig. 7E

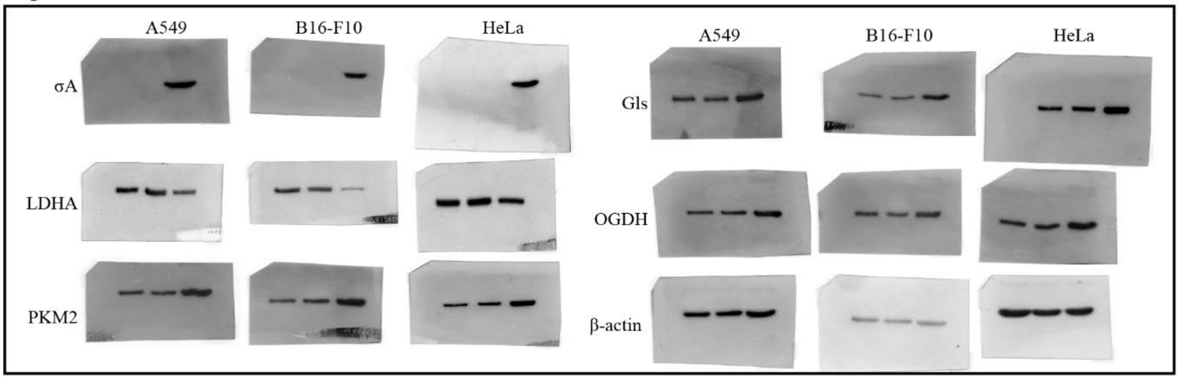

Fig. S4

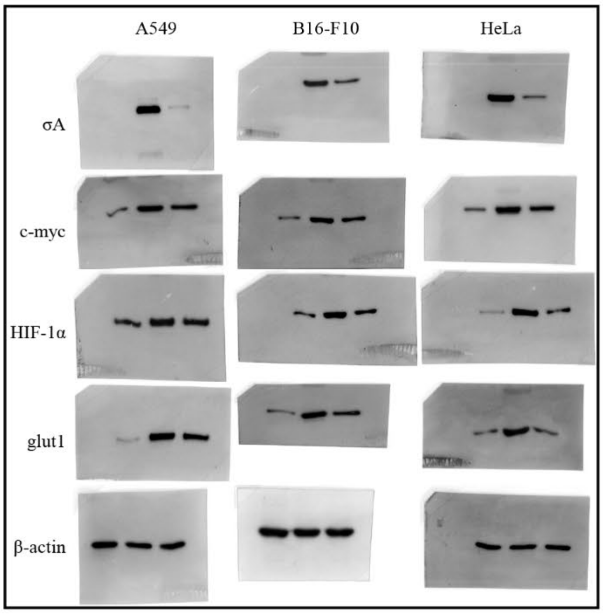

Fig. S5

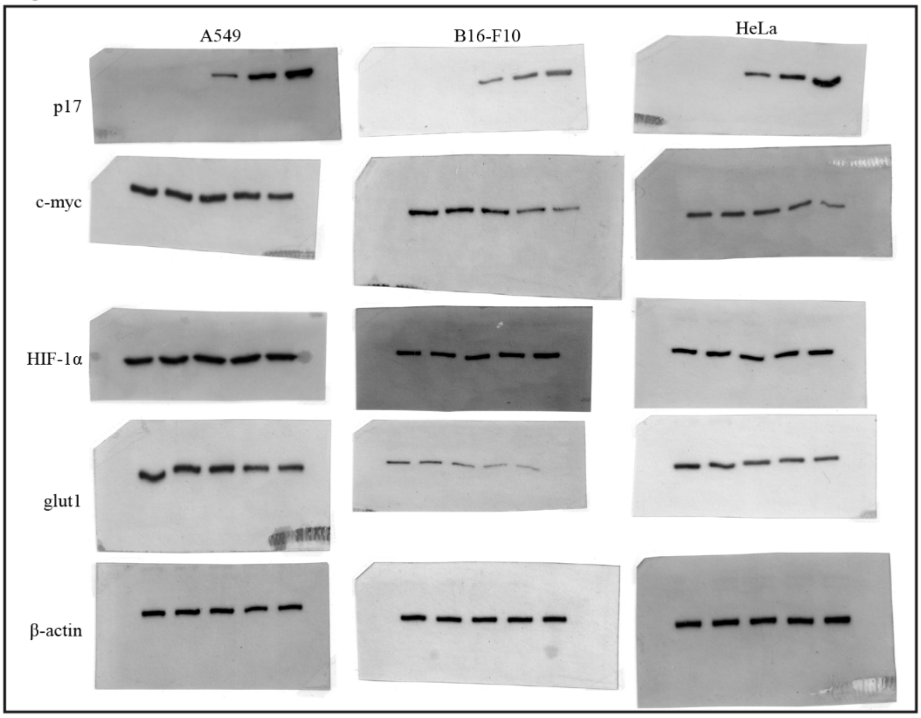

Fig. S6 A

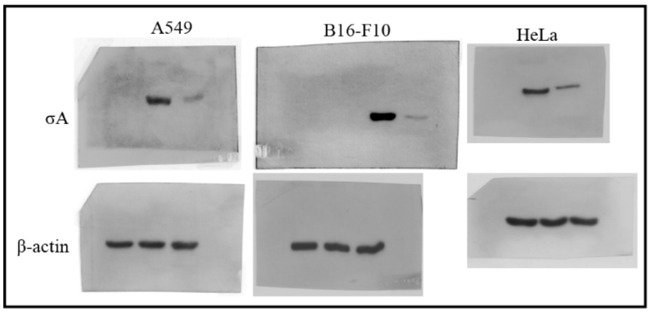

Fig. S6 B

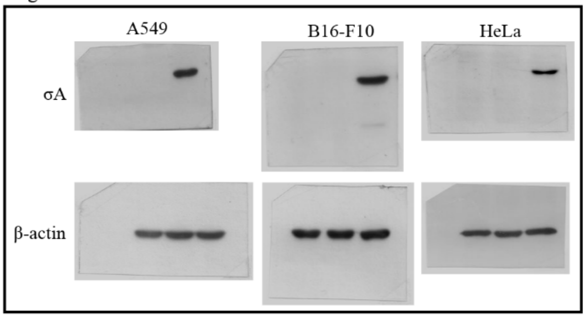

Supplement: Supplementary file 1 [file viruses-15-00523-s001.zip › viruses-2194336-supplementary.pdf]
